# Mutation to *ispA* Produces Stable Small-Colony Variants of Pseudomonas aeruginosa That Have Enhanced Aminoglycoside Resistance

**DOI:** 10.1128/aac.00621-22

**Published:** 2022-07-05

**Authors:** Melissa Pitton, Simone Oberhaensli, Fiona Appiah, Jean-Luc Pagani, Anne Fournier, Stephan M. Jakob, Yok-Ai Que, David R. Cameron

**Affiliations:** a Department of Intensive Care Medicine, Inselspital, Bern University Hospital, University of Bern, Bern, Switzerland; b Graduate School for Cellular and Biomedical Sciences (GCB), University of Bern, Bern, Switzerland; c Interfaculty Bioinformatics Unit, University of Bern, Bern Switzerland; d SIB Swiss Institute of Bioinformatics, Lausanne, Switzerland; e Service of Intensive Care Medicine, Lausanne University Hospital and University of Lausanne, Lausanne, Switzerland; f Service of Pharmacy, Lausanne University Hospital and University of Lausanne, Lausanne, Switzerland

**Keywords:** SCV, antibiotic resistance, tobramycin, gentamicin, burn wound infection, *Pseudomonas aeruginosa*, small-colony variant, aminoglycosides

## Abstract

Pseudomonas aeruginosa is a major pathogen in burn wound infections. We present one of the first reports of small-colony variant (SCV) emergence of P. aeruginosa, taken from a patient under aminoglycosides for a persistent burn wound infection. We confirm the causative role of a single *ispA* mutation in SCV emergence and increased aminoglycoside resistance. IspA is involved in the synthesis of ubiquinone, providing a possible link between electron transport and SCV formation in P. aeruginosa.

## INTRODUCTION

Pseudomonas aeruginosa is an opportunistic pathogen capable of establishing infections that are difficult to eradicate with antibiotics, and it remains the most frequent Gram-negative microorganism isolated from burn wounds (1). The refractory nature of P. aeruginosa during infection is often associated with the evolution toward host-adapted phenotypes, including biofilm production, high persister variants, conversion to mucoidy, altered expression of virulence factors, and the formation of small-colony variants (SCVs) (2–5). SCVs are characterized by their small colony size on agar plates, slow growth rate, and atypical metabolism (6). SCVs have been frequently associated with persistent and antibiotic-resistant infections caused by P. aeruginosa and other opportunistic pathogens, including Staphylococcus aureus (7), making them an important target for the development of future therapies.

While the mechanisms of SCV formation in P. aeruginosa appear diverse, often involving the secondary messenger cyclic di-GMP (8, 9) or global changes in gene expression (10, 11), SCV formation in S. aureus is comparatively conserved, with archetypal strains auxotrophic for hemin, menadione, and/or thymidine (12, 13). Heme and menadione are involved in the production of cytochrome and menaquinone, respectively, linking S. aureus SCVs to dysfunctional electron transport (14). A link between electron transport and SCV formation for P. aeruginosa, however, is yet to be described.

**FIG 1:**
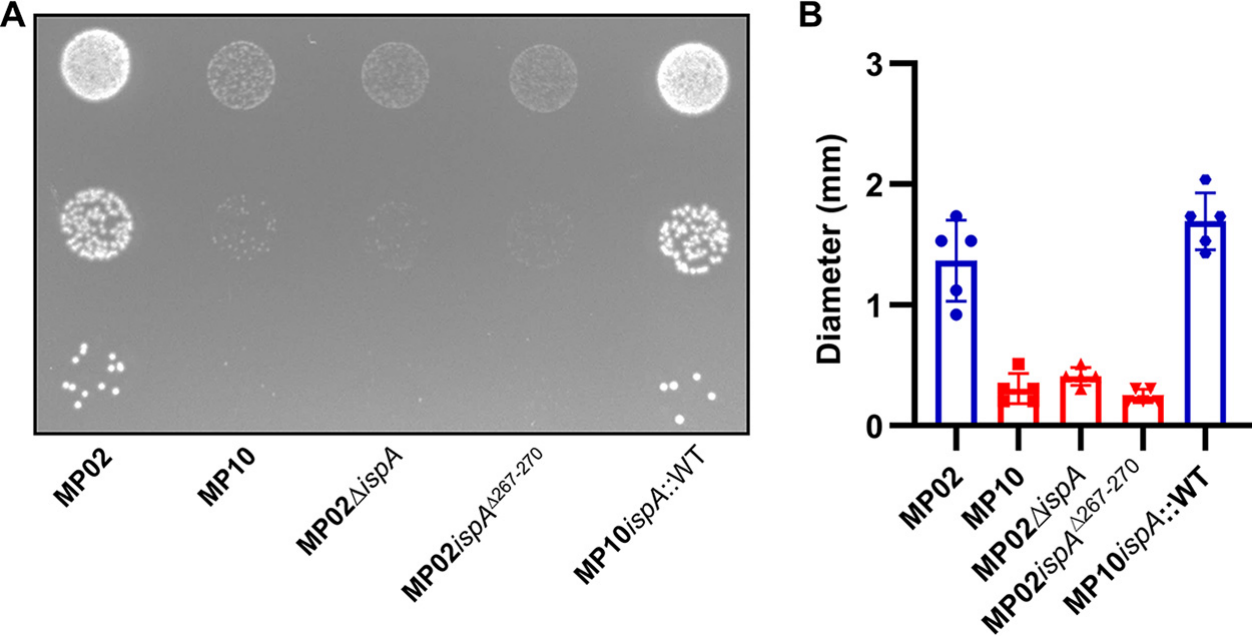
Colony sizes of clinical P. aeruginosa isolates and engineered *ispA* mutants. (A) Overnight suspensions were 10-fold serially diluted. We plated 10-μL of dilutions 10^−5^, 10^−6^, and 10^−7^ on LB agar. Colonies were formed for 30 h at 37°C. MP02 is a normal colony variant (NCV), and MP10 is a small-colony variant (SCV). (B) Colony size quantification (*n* = 6). NCVs are blue, and SCVs are red.

In the current study, we performed comparative genomics for a P. aeruginosa SCV and its “normal” colony counterpart (NCV). P. aeruginosa clinical isolates MP02 and MP10 were collected from the same sample taken from a hospitalized patient with severe burn wounds (see Table S1 in the supplemental material) (15). Need for informed consent and authorization for analyzing the previously collected bacterial isolates was waived by the local ethical committee. MP02 was an NCV, whereas MP10 was an SCV that produced smaller colonies on solid agar (Fig. 1A). Compared with MP02, MP10 also had a modest growth defect when grown in Luria-Bertani (LB) medium (Fig. S1). MP10 was serially propagated five times in liquid media, and colony sizes remained small when plated on agar, suggesting the phenotype was nontransient.

Prior to isolation of MP02 and MP10, the patient had been treated with a range of antibiotics, including tobramycin, which was applied after the emergence of extensive antibiotic resistance. MP02 was defined in the clinic as intermediate susceptible to tobramycin, as its minimum inhibitory concentration (MIC) was at the susceptibility breakpoint using European Committee on Antimicrobial Susceptibility Testing (EUCAST) guidelines (2 μg/mL) (16). Conversely, MP10 was classified as resistant, with an Etest (bioMérieux, Switzerland) MIC of 4 μg/mL. We acquired the isolates and performed additional antibiotic susceptibility testing using broth microdilution according to Clinical and Laboratory Standards Institute guidelines and confirmed that MP10 had a modest but reproducible 2-fold increase in MIC for aminoglycosides tobramycin, gentamicin, and amikacin (17) (Table 1).

**TABLE 1:** Aminoglycoside MICs as determined per CLSI guidelines using the microdilution method in Mueller-Hinton broth^*a*^

Strain	Characteristic	MIC (μg/mL) of:		
		Tobramycin	Gentamicin	Amikacin
MP02	Clinical normal colony variant	4	32	32
MP10	Clinical small-colony variant	8	64	64
MP02Δ*ispA*	In-frame deletion *ispA* mutant	8	64	64
MP02*ispA*^Δ267-270^	MP02 engineered with the 12-base-pair deletion found in MP10	8	64	64
MP10*ispA*::WT	MP10 complemented in *cis* with wild-type *ispA*	4	32	32

a Values were determined following 18 hours of incubation at 37°C. Experiments were performed three times in duplicates to confirm and ensure reproducibility. WT, wild-type; CLSI, Clinical and Laboratory Standards Institute.

**FIG 2:**
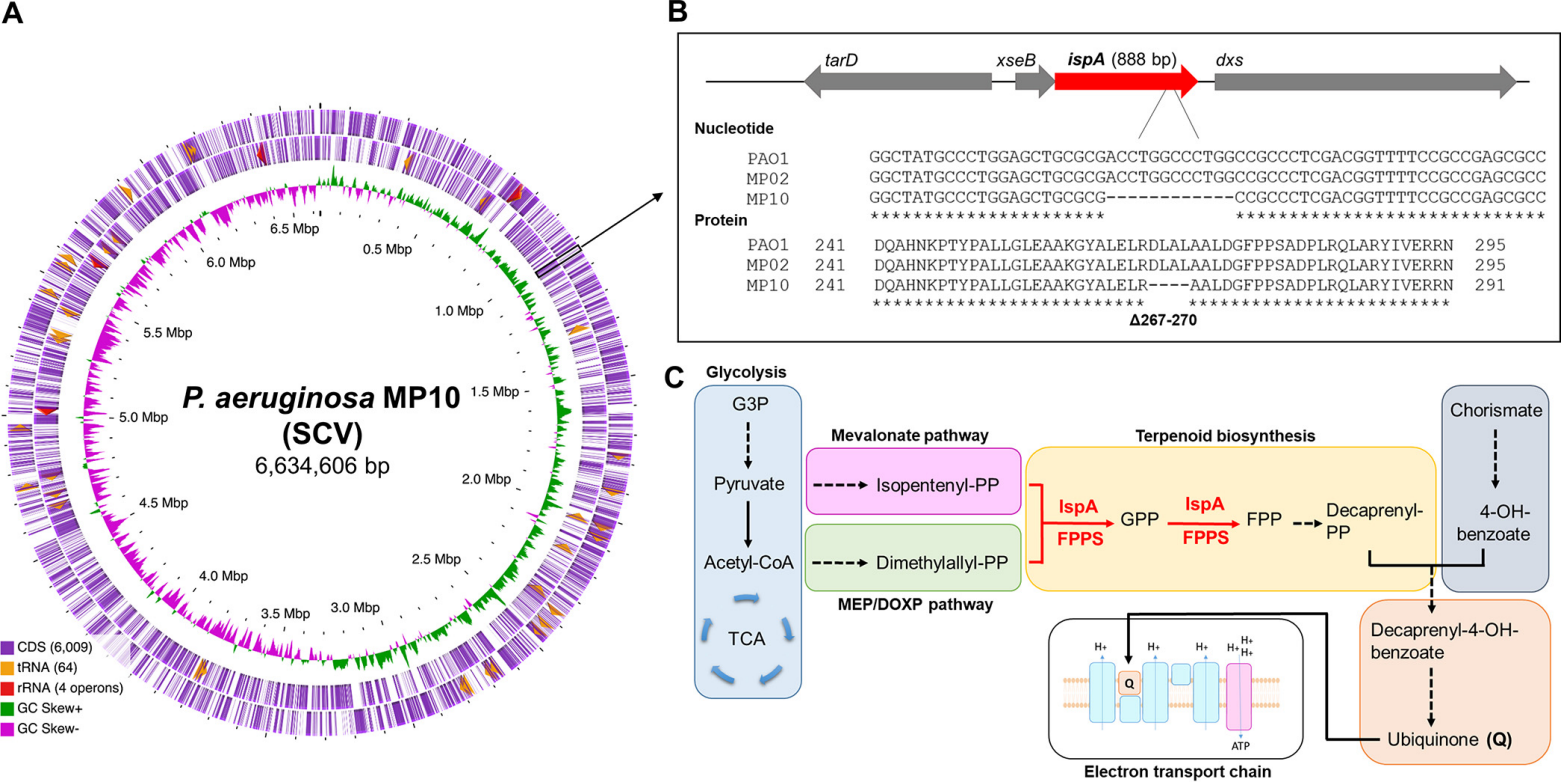
*ispA* genomic landscape. (A) Circular genome representation of MP10 (SCV) created using CGView (32). The inner ring illustrates the GC skew. The outer two rings represent coding sequences (CDS), tRNAs, and rRNAs on the reverse and forward strands, respectively. A black box is included to highlight the *ispA* gene. (B) Genomic localization of *ispA*. Nucleotide and protein alignments of PAO1 (GenPept accession number NP_250121), MP02, and MP10, showing the 12-bp deletion in MP10 and the consequent deletion of amino acids from 267 to 270. Protein and nucleotide sequence alignments were generated using Clustal Omega (33). (C) Schematic pathway displaying the involvement of IspA (also knowns as FPPS) in ubiquinone biosynthesis. Dotted arrows indicate that multiple steps have been abbreviated. FPP, farnesyl pyrophosphate; FPPS, farnesyl pyrophosphate synthase; G3P, glycerol-3-phosphate; GPP, geranyl pyrophosphate; MEP/DOXP, 2-*C*-methyl-d-erythritol 4-phosphate/1-deoxy-d-xylulose 5-phosphate; PP, pyrophosphate; TCA, tricarboxylic acid.

Complete, circular genome sequences of MP02 and MP10 were resolved using PacBio reads that were assembled using Flye (18) and then polished using Illumina sequencing reads (19). Each genome was annotated using the Prokaryotic Genome Annotation Pipeline (PGAP) (20) and deposited at DDBJ/ENA/GenBank (accession numbers CP063394 and CP063393, respectively). MP10 had a genome of 6,634,606 bp, average GC content of 66%, and 6,136 coding DNA sequences (CDS) (Fig. 2A). Comparative genome analysis at single nucleotide resolution identified only one mutation between MP10 and MP02; MP10 possessed a 12-bp deletion in the *ispA* gene, which codes for farnesyl pyrophosphate synthase (FPPS) (Fig. 2B). FPPS catalyzes the reaction required to generate geranyl and farnesyl pyrophosphate (GPP and FPP), both of which are substrates involved in the biosynthesis of the electron carrier ubiquinone (Fig. 2C). The mutation resulted in an in-frame deletion of four amino acids (from positions 267 to 270).

We reasoned that the SCV phenotype and increased resistance to aminoglycosides of MP10 were caused by the *ispA* mutation. We generated *ispA* mutants by bidirectional allelic exchange using the method of Hmelo et al. (21) with PCR primers listed in Table S3. The *ispA* mutant allele (*ispA*^Δ267-270^) was engineered into the NCV strain MP02 generating MP02*ispA*^Δ267-270^, and the full-length *ispA* allele (*ispA*::WT) was introduced into MP10 to complement in *cis* the mutation, generating strain MP10*ispA*::WT. Additionally, full-length *ispA* was deleted from MP02 (MP02Δ*ispA*) (Table S1).

**FIG 3:**
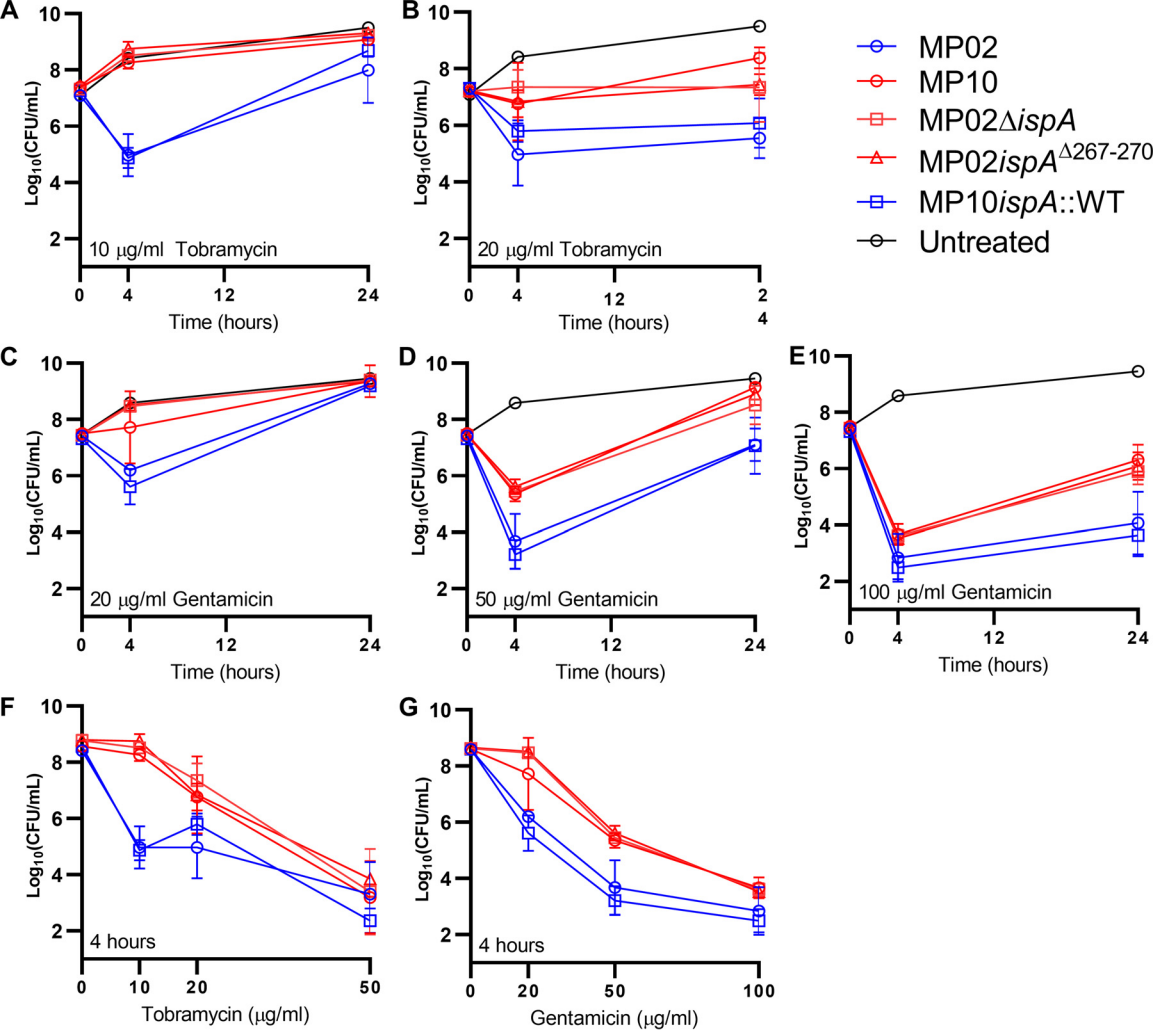
Mutation to *ispA* results in decreased aminoglycoside killing. Bacteria were grown to mid-exponential phase (~2 × 10^7^ CFU/mL) in LB at 37°C and then treated with different concentrations of tobramycin or gentamicin. Time-dependent (A to E) and concentration-dependent (F and G) curves are shown. Normal colony variants are blue; small-colony variants are red. The limit of detection was 10^2^ CFU/mL.

Strains with full-length *ispA* displayed normal colony size, whereas strains with mutated *ispA* were SCVs (Fig. 1). Additionally, P. aeruginosa with mutated *ispA* had 2-fold-higher tobramycin, gentamicin, and amikacin MICs than strains with full-length *ispA* (Table 1). To delineate the possible impact of this modest increase in MIC on treatment efficiency, we performed *in vitro* antibiotic killing assays as described elsewhere (22). NCVs and SCVs were grown in LB media at 37°C for 3 h and 4 h, respectively, to reach the same concentration of exponentially growing cells (~2 × 10^7^ CFU/mL) prior to the addition of antibiotics. At 10 μg/mL tobramycin, which is close to the peak serum levels for burn patients treated with extended-interval tobramycin (7.4 μg/mL; range, 3.1 to 19.6) (23), >99.9% of exponentially growing MP02 cells were killed following 4 h of incubation (Fig. 3A). In contrast, MP10 grew similarly to the untreated control over a 24-h period, confirming that the difference in antibiotic susceptibility was due to enhanced resistance as opposed to enhanced tolerance (using definitions from reference 24). MP02 showed evidence of regrowth after 24 h of incubation, and this was accompanied by a 4-fold increase in MIC (4 to 16 μg/mL) for the surviving colonies. At 20 μg/mL, the emergence of resistance for MP02 was suppressed at 24 h (Fig. 3B). We performed tobramycin time-killing assays in a second medium, M9 minimal medium supplemented with 20 mM glucose, and produced similar results (Fig. S2). Time-kill data were also similar when using a second antibiotic from the aminoglycoside class, gentamicin (Fig. 3C and D); however, a higher concentration (100 μg/mL) was required to suppress resistance emergence for MP02 (Fig. 3E). Focusing on the 4-h time point, compared with MP02, the survival of MP10 was significantly higher following treatment with tobramycin 10 μg/mL and 20 μg/mL (~2,000-fold, *P* < 0.0001, and ~60-fold, *P* = 0.0002, respectively; two-way analysis of variance [ANOVA] with multiple comparisons using the method of Dunnett; Fig. 3F). No difference in survival was determined at 50 μg/mL. Similarly, compared with MP02, survival of MP10 was significantly higher at 20 μg/mL, 50 μg/mL, and 100 μg/mL of gentamicin (~30-fold, *P* < 0.0001, ~50-fold, *P* < 0.0001; and ~7-fold, *P* < 0.05, respectively; Fig. 3G). Likewise, at the 24-h time point, MP10 showed statistically higher survival than MP02 following treatment with tobramycin and gentamicin (*P* < 0.01 and *P* < 0.0001, respectively, two-way ANOVA with Dunnett’s test; two-way ANOVA; Fig. S3).

Concentration-dependent killing and time-dependent killing phenotypes of engineered mutants were determined by the *ispA* allele. Strains with full-length *ispA* revealed phenotypes similar to MP02, and those with mutated *ispA* (either deletion or the clinical variant, *ispA*^Δ267-270^) were similar to MP10, confirming the causative role for *ispA* mutation in reduced aminoglycoside susceptibility (Fig. 3).

IspA is conserved across diverse bacteria. Disruption of *ispA* reduced growth yield in Escherichia coli (25), reduced spreading for Shigella flexneri (26), and produced an SCV-like phenotype in laboratory-generated mutants of S. aureus (27). Further, aminoglycoside exposure *in vitro* produced *ispA* mutants of E. coli and P. aeruginosa PA14 that had enhanced gentamicin resistance (28, 29) suggesting *ispA* may be a broad evolutionary target for SCV formation and/or reduced susceptibility to aminoglycosides.

IspA is a key enzyme in the synthesis of the electron carrier ubiquinone; E. coli *ispA* mutants that evolved *in vitro* had limited ubiquinone pools (30), and strains engineered to overexpress *ispA* produced more ubiquinone (31). Dysfunctional electron transport is a frequently described mechanism of SCV formation for S. aureus; the current report provides the first evidence linking SCV formation to electron transport for P. aeruginosa. Future studies are warranted to determine the effect of *ispA* mutation upon bioenergetics and to determine whether this is a convergent mechanism across diverse bacterial pathogens.

### Data availability.

Genomes of MP02 and MP10 were deposited into DDBJ/ENA/GenBank (accession numbers CP063394 and CP063393, respectively).
