# Supplementary material for: Mutation to ispA Produces Stable Small-Colony Variants of Pseudomonas aeruginosa That Have Enhanced Aminoglycoside Resistance
Source: Antimicrob Agents Chemother. 2022 Jul 5;66(7):e00621-22. doi: 10.1128/aac.00621-22 (PMC9295567; doi:10.1128/aac.00621-22)
Supplement: Supplemental file 1 — Tables S1 to S3 and Fig. S1 to S3. Download aac.00621-22-s0001.pdf, PDF file, 0.5 MB [file aac.00621-22-s0001.pdf]

SUPPLEMENTAL MATERIAL

Mutation to *ispA* produces stable small colony variants of *Pseudomonas aeruginosa* that have enhanced aminoglycoside resistance

Melissa Pitton[1,2], Simone Oberhaensli[3], Fiona Appiah[1], Jean Luc Pagani[4], Anne Fournier[4], Stephan M. Jakob[1], Yok-Ai Que[1,#,*], and David R. Cameron[1,*]

1. Department of Intensive Care Medicine, Inselspital, Bern University Hospital, University of Bern, Bern, Switzerland
2. Graduate School for Cellular and Biomedical Sciences (GCB), University of Bern, Bern, Switzerland
3. Interfaculty Bioinformatics Unit and SIB Swiss Institute of Bioinformatics, University of Bern, Bern, Switzerland
4. Service of Intensive Care Medicine, Lausanne University Hospital and University of Lausanne, Lausanne, Switzerland

***** equal contribution

#Corresponding author

Yok-Ai Que, MD, PhD
Department of Intensive Care Medicine, INO E-403
Inselspital; Bern University Hospital
3010 Bern, Switzerland
Tel: +41 31 632 45 21
E-Mail: Yok-Ai.Que@insel.ch

Keywords: SCV, antibiotic resistance, tobramycin, gentamycin, burn wound infection.

Table S1. Strains and plasmids used in this study

**Strains/Plasmids**	**Description**	**Phenotype**	**Source**
P. aeruginosa			
MP02	Clinical isolate	NCV	This study
MP10	Clinical isolate	SCV	This study
MP02Δ*ispA*	MP02 mutant with *ispA* in-frame deletion	SCV	This study
MP02*ispA*$^{\Delta 267-270}$	MP02 mutant with *ispA* harboring the 12-base pair deletion identified in MP10	SCV	This study
MP10*ispA*::WT	MP10 mutant complemented in *cis* with wild-type *ispA*	NCV	This study
E. coli			
DH5α	Cloning strain		Lab stock
SM10λpir	Conjugative strain for biparental mating		(1)
**Plasmids**			
pEX18Gm	Gene replacement vector, oriT+, sacB+, MCS from pUC18, GmR (=gentamicin resistance)		(2)
pEXΔ*ispA*	pEX18 derivative used to delete *ispA*		This study
pEX*ispA*$^{\Delta 267-270}$	pEX18 derivative used to produce *ispA*$^{\Delta 267-270}$		This study
pEX*ispA*::WT	pEX18 derivative used to introduce wild-type *ispA*		This study

Abbreviations: NCV, normal colony variant; SCV, small colony variant.

Table S2. Antibiograms for MP02 (NCV) and MP10 (SCV)

Antibiotics	MP02	MP10
piperacillin/tazobactam	R	R
ceftazidime	R	R
cefepime	R	R
imipenem	R	R
meropenem	R	R
aztreonam	R	R
amikacin	R	R
gentamicin	R	R
tobramycin	I	R
colistin	S	S
trimethoprim/sulfamethoxazole	R	R
ciprofloxacin	R	R
levofloxacin	R	R

NCV, normal colony variant; R, resistant; I, intermediate; S, susceptible; SCV, small colony variant
Susceptibility profiles as determined by the Institute of Microbiology of the University of Lausanne.

Table S3. Primers used for mutagenesis

**Name**	**Sequence (5' → 3')**	**Characteristics**
ispA_up_fwd	ccctctaga**ccggcgtaacgggaaagg**	Forward primer to delete full-length *ispA* gene (upstream region)
ispA_up_rev	*agttgcgtcgct***gatcatgcctcgtcgccttc**	Reverse primer to delete full-length *ispA* gene (upstream region)
ispA_down_fwd	**gacgcaactgaccgacgg**	Forward primer to delete full-length *ispA* gene (downstream region)
ispA_down_rev	cccgaattc**gctcggtgaggatcttgtgc**	Reverse primer to delete full-length *ispA* gene (downstream region)
ispA_267-270_del_fwd	ccctctaga**ggaacatgccgtctgtctgg**	Forward primer to delete amino acids from 267 to 270
ispA_267-270_del_rev	cccgaattc**ggatcttgtgcggataggcc**	Reverse primer to delete amino acids from 267 to 270
ispA_ins_fwd	**cgcatcatcaggtcccagg**	Forward primer to confirm mutagenesis
ispA_ins_rev	**ggtgtcgtactcgctctctg**	Reverse primer to confirm mutagenesis

Regions of homology to the target amplicons are in **bold**, regions of reverse complementarity are *italicized* and restriction sites are underlined.

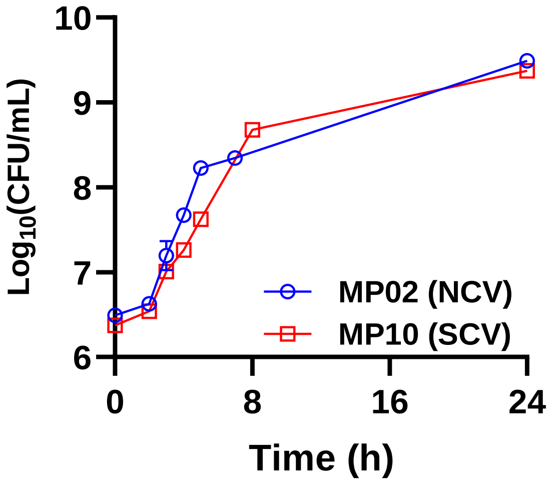

Figure S1. Growth of MP02 and MP10 in LB media at 37°C with constant shaking. CFU, colony formatting unit; NCV, normal colony variant; SCV, small colony variant.

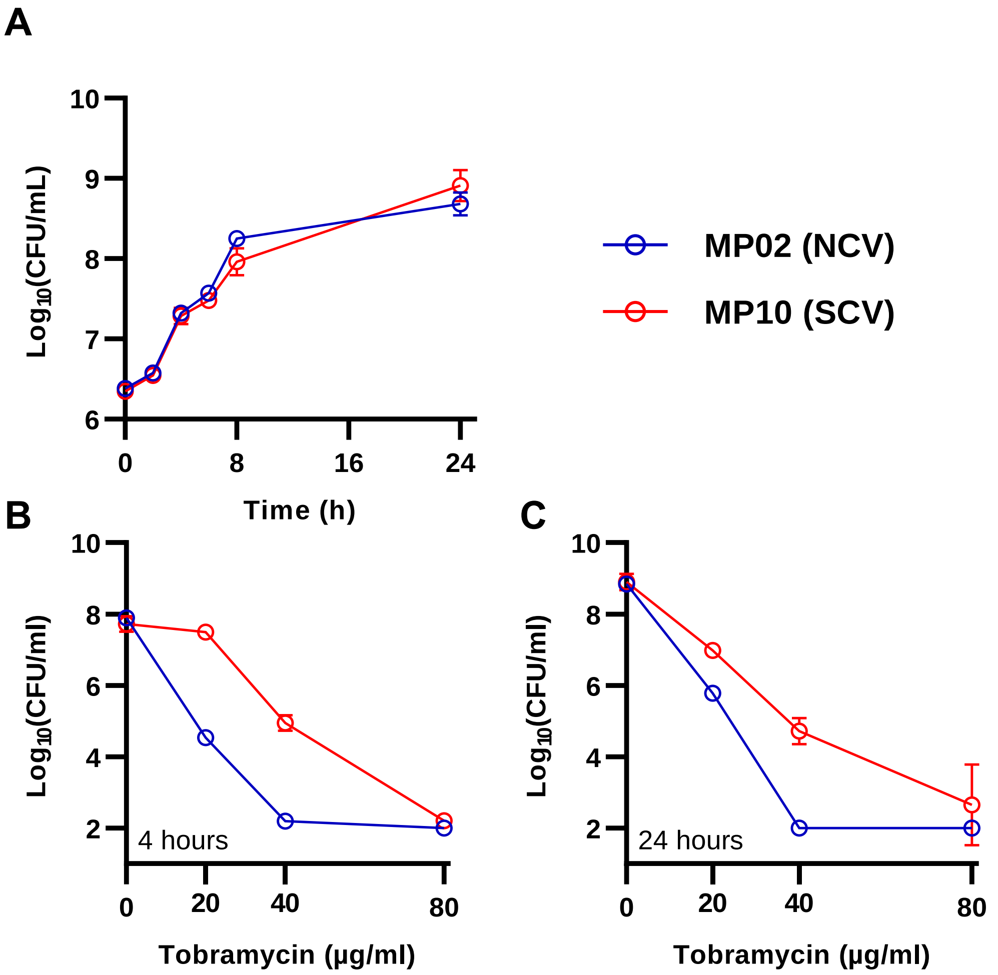

Figure S2. Mutations in *ispA* decrease tobramycin-induced killing in M9 minimal medium supplemented with 20 mM glucose. (A) Growth curves of MP02 and MP10 incubated in M9 medium at 37°C under constant shaking. **(B, C)** Bacteria were grown to mid-exponential phase in M9 at 37°C before treatment for 4h **(B)** or 24h **(C)** with increasing concentrations of tobramycin. CFU, colony formatting unit; NCV, normal colony variant; SCV, small colony variant.

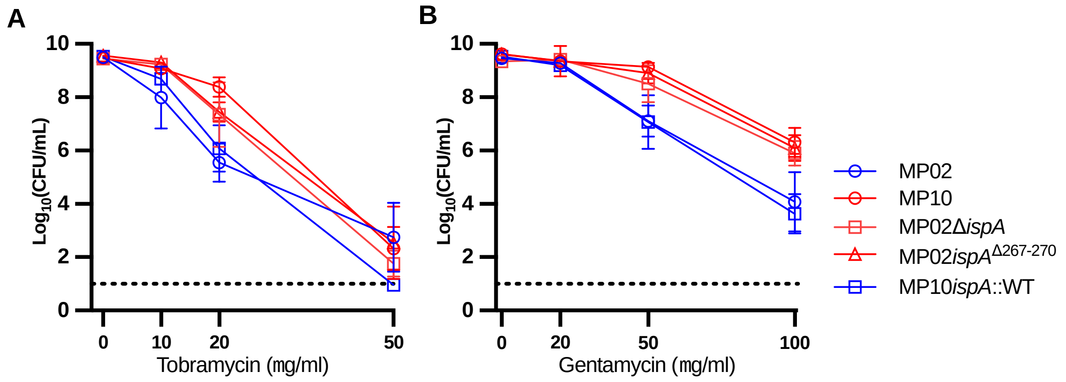

Figure S3. Mutations in *ispA* decrease aminoglycoside-induced killing in LB media. Bacteria were grown to mid-exponential phase in LB at 37°C and then treated for 24h with either tobramycin **(A)** or gentamicin **(B)**. CFU, colony formatting unit; NCV, normal colony variant; SCV, small colony variant.

REFERENCES

1. Miller VL, Mekalanos JJ. A novel suicide vector and its use in construction of insertion mutations: osmoregulation of outer membrane proteins and virulence determinants in *Vibrio cholerae* requires *toxR*. *Journal of bacteriology* 1988; 170: 2575-2583.

2. Hoang TT, Karkhoff-Schweizer RR, Kutchma AJ, Schweizer HP. A broad-host-range Flp-FRT recombination system for site-specific excision of chromosomally-located DNA sequences: application for isolation of unmarked *Pseudomonas aeruginosa* mutants. *Gene* 1998; 212: 77-86.
